# Edge Doping Engineering of High-Performance Graphene Nanoribbon Molecular Spintronic Devices

**DOI:** 10.3390/nano12010056

**Published:** 2021-12-26

**Authors:** Haiqing Wan, Xianbo Xiao, Yee Sin Ang

**Affiliations:** 1 Department of Ecology and Environment, Yuzhang Normal University, Nanchang 330029, China; 2 Science, Mathematics and Technology (SMT), Singapore University of Technology and Design, Singapore 487372, Singapore; 3 School of Computer Science, Jiangxi University of Chinese Medicine, Nanchang 330004, China; 20101034@jxutcm.edu.cn

**Keywords:** density functional theory, graphene spintronics, quantum transport

## Abstract

We study the quantum transport properties of graphene nanoribbons (GNRs) with a different edge doping strategy using density functional theory combined with nonequilibrium Green’s function transport simulations. We show that boron and nitrogen edge doping on the electrodes region can substantially modify the electronic band structures and transport properties of the system. Remarkably, such an edge engineering strategy effectively transforms GNR into a molecular spintronic nanodevice with multiple exceptional transport properties, namely: (i) a dual spin filtering effect (SFE) with 100% filtering efficiency; (ii) a spin rectifier with a large rectification ratio (RR) of 1.9 ×$10^{6}$; and (iii) negative differential resistance with a peak-to-valley ratio (PVR) of 7.1 ×$10^{5}$. Our findings reveal a route towards the development of high-performance graphene spintronics technology using an electrodes edge engineering strategy.

## 1. Introduction

Nanostructures and nanodevices with unusual spin-transport properties, such as dual spin filtering [1,2], magnetoresistance effect [3], molecular rectification [4] and negative differential resistance (NDR) [5], have attracted tremendous research interests in recent years due to their enormous potential as the building blocks in spintronics technology. Two-dimensional (2D) materials, such as graphene, have been extensively studied due to their exceptional physical and transport properties [6,7,8,9]. In addition, they will play an important role in a large variety of solid-state device applications, such as nanoelectronics [10], spintronics [11], valleytronics [12], nonlinear photonics [13], optoelectronics [14] and biochemical sensors [15].

Quasi one-dimensional graphene nanoribbons (GNRs) represent a technologically important extension of 2D nanosheets. GNRs can be tailored from monolayer graphene [16,17] and the controlled formation of GNRs has been demonstrated experimentally using a transmission electron microscope-scanning tunneling microscope system [18]. Because of the existence of edge states around the Fermi level, the electronic and transport properties of GNRs are sensitively influenced by their edge morphology, thus opening up a versatile edge engineering avenue for designing high-performance field-effect transistors [19], spintronics and neuromorphic devices [20].

Zigzag-edged graphene nanoribbons (ZGNRs) are particularly promising due to the presence of electric field effect tuning of the spin-polarized edge states [20,21]. The ZGNRs can be further designed into nanodevices with unusual transport behaviors, such as thermal regulation [22], spin filtering [7], spin diode [21] and NDR [23] effects, using a plethora of strategies, including edge modifications [24,25], doping [26,27] and a applying magnetic field [28]. ZGNRs thus represent a versatile *designer* platform to explore the design of high-performance spintronics devices with novel functionalities. Particularly, the stitching of multiple zigzag-edged, triangular-shaped graphene nanoflakes into a sawtooth GNR (STGNR) nanodevice allows for the generation of ferromagnetic metal, half-metal or a bipolar magnetic semiconductor [24] or spin semiconductor [25] depending on the geometry and the application of an external electrical field. Although hybridization may lead to coupled modes with little dispersion and very low Fermi velocity, the Fermi velocity can be tuned by applying gate voltages [29]. It has also been demonstrated that the sub-6 nm-wide GNRs offer approximately 45 cm${}^{2}$/Vs at the bandgap of ∼0.1 eV, while edge defects may severely limit GNR-based devices due to lower electrical mobility [30].

The design of GNR-based spintronics devices can be further enhanced via edge doping. A GNR with edge doping is stable and can be manipulated in terms of its spin transport properties [26]. Recently, N-doped and B-doped GNRs have been successfully synthesized experimentally [31,32], and theoretically predicted that the doping positions of B and N atoms play a significant role in the electronic band structure of the edge transport channels [27]. In relevance to the design of spintronic devices, a perfect bipolar spin filtering effect can be achieved by manipulating the doping positions of B or N atoms [33]. Moreover, B/P-doped GNRs are also predicted to exhibit an NDR effect [34], which is a potential building block for memory devices, oscillators and fast-switching devices.

The electronic band structures and the spin filtering effect of ZGNRs have previously been investigated [23,33]. In a pristine STGNR-based device, the spin-polarized current transport can be realized with antiparallel (AP) spin ordering of the two electrodes, while in the parallel (P) configuration, the electron tunneling channels for both α- and β-spin states are almost blocked off completely [21]. Importantly, as the spin-polarized transport occurs along the STGNR edges [25], the edge engineering can thus be harnessed to control the spin transport of the nanodevice. Nevertheless, the spintronic quantum transport properties of B/N-doped STGNRs remains largely incomplete thus far.

In this work, we carry out first-principles calculations to investigate the spintronic transport properties of STGNRs co-coped with B and N in the electrode regions. Our results show that the B/N edge doping provides an efficient tool to tune the electronic band structures of STGNRs which sensitively influences the spintronic transport properties of a source–channel–drain nanodevice structures. Interestingly, our proposed structures exhibit exceptional transport behaviors, including the 100% spin-filtering effect (SFE), spin rectification with a rectification ratio (RR) of 1.9 ×$10^{6}$ and a negative NDR effect with a peak-to-valley ratio (PVR) of 7.1 × 10${}^{5}$. Our findings concretely establish the edge-doping strategy as a potential promising route towards the design and development of STGNR-based nanodevices, such as molecular spin diode, random access memory cells and fast switching devices.

## 2. Computational Details

**Figure 1:**
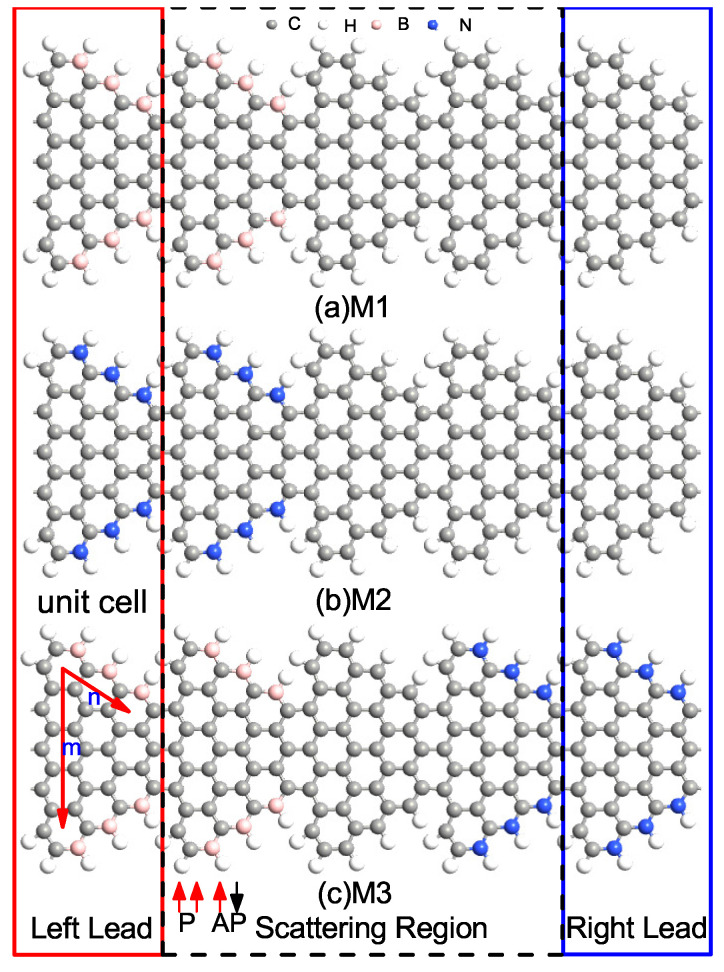
(Color online) The constructed device models for edge-doped STGNRs (m, n), where M1, M2 and M3 correspond to (**a**) left B-doped and right pristine STGNR electrode, (**b**) left N-doped and right pristine STGNR electrode and (**c**) left B-doped and right N-doped electrodes. The black, grey, pink and blue spheres represent the C, H, B, and N atoms, respectively. The red/blue frame indicates the unit cell of left/right electrode, and (m, n). The red/black arrow indicates the spin-up (α-spin)/spin-down (β-spin) states in the electrodes, and P/AP configuration indicates the spin of the left and right electrode is parallel/antiparallel directions, respectively.

We consider a two terminal source–channel–drain nanodevice as shown in Figure 1. The STGNRs (m,n) device is divided into three regions: left electrode (bounded by red frame in Figure 1), right electrode (bounded by blue frame in Figure 1) and the scattering region (bound by dashed black frame in Figure 1). The integer (m,n) denotes the number of hexagonal rings along the width and the length of a nanoribbon in a unit cell, respectively. The edge atoms are saturated by 1H atoms as 1H termination (sp${}^{2}$ hybridization) is commonly employed in both pristine and doped GNRs [35]. We investigated STGNR nanodevices with three different electrode combinations as shown in Figure 1: (i) device M1 with B-doped left electrode and pristine STGNR right electrode, (ii) device M2 with N-doped left electrode and pristine STGNR right electrode and (iii) device M3 with B-doped left electrode and N-doped STGNR right electrodes. The scattering region contains three unit cells. The left cell belongs to the left electrode, and the right cell belongs to the right electrode. We have checked the spin-dependent transmission spectra of the devices with different scattering region lengths and found that they are almost identical, thus suggesting that the chosen structures are sufficient in screening the interaction between the electrodes and the center scattering channel region. Since STGNR (5, 3) is a bipolar magnetic semiconductor [21] and edge doping can manipulate its spin transport properties, the pristine and edge-doped STGNR (5, 3) are adopted as the electrodes of the device. The left electrode can be switched between the P and AP magnetization configurations with respect to the right electrode. The scattering region includes a unit cell of pristine STGNR (5, 3). The electrodes used in our model are ideal contacts as a part of the central region, and the contact regions have an identical crystal and electronic band structure. It should be noted that when contacted by an external metal, the electronic properties of the contact heterostructure could be significantly modified due to the coupling and interaction between the metal contact and the GNR [9,36,37]. High-quality electrical contacts with weak van der Waals coupling [38,39,40] with the GNR are thus required to preserve the spintronic transport properties predicted in this work.

The geometry optimization and electron transport calculations for each system are performed by ab initio software package, Atomistix ToolKit [41,42]. The geometry optimization for each system is used by a quasi-Newton method [43] until the forces on each atom is <0.05 eV/Å. The electron transports are calculated by density functional theory combined with nonequilibrium Green’s function method. The self energy matrix is calculated from the computationally efficient Sancho–Rubio method [44]. The Perdew–Zunger parameterization of the local spin density approximation is used for the exchange correlation functional [45]. Double-ζ plus polarization basis set is adopted for all of the atoms. In the transport calculation, the k-point sampling is 1 × 1 × 100, the energy cutoff is 150 Ry and electronic temperature is set to 300 K. The spin-resolved current(I${}_{\sigma }$) is calculated by the Landauer–Büttiker formula [46]:

(1) $$I_{\sigma }\left(V_{b}\right)=\frac{e}{h}\int_{-\infty }^{\infty }T_{\sigma }\left(E,V_{b}\right)\left[f_{L}\left(E-\mu_{L}\right)-f_{R}\left(E-\mu_{R}\right)\right]dE,$$

where σ=α,β denotes the two spin polarization, $\mu_{L/R}$ is the electrochemical potential, $f_{L/R}\left(E\right)$ is the Fermi distribution function of the left/right electrode, $T_{\sigma }\left(E,V_{b}\right)$ is the transmission spectrum at energy *E* and at bias potential $V_{b}$.

## 3. Results and Discussions

### 3.1. Spin-Resolved Tunneling Current, Rectification Ratio and Negative Differential Resistance Effect

**Figure 2:**
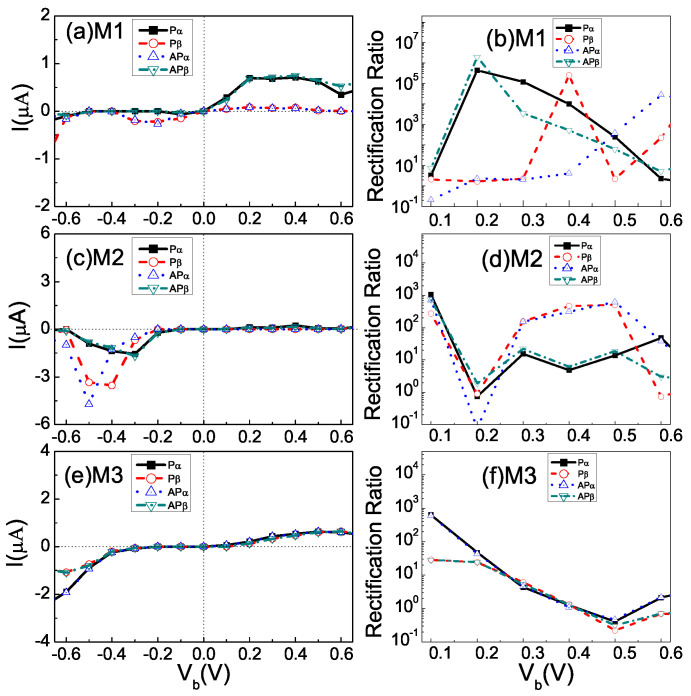
(Color online) Calculated spin-resolved I-V curves and rectifying ratio (RR) with the P and AP configuration, (**a**,**b**) for M1 structure, (**c**,**d**) for M2 and (**e**,**f**) for M3, respectively.

Figure 2 presents the spin-resolved *I*-*V* curves and rectifying ratio with the P and AP configuration within a bias range from −0.6 V to +0.6 V for the devices M1–M3. Several important features are clearly visible: Firstly, the device M1 shows dual spin filter efficiency. As shown in Figure 2a, for device M1, the current of the P${}_{\alpha }$ /AP${}_{\beta }$ electron in the positive bias region is obviously larger than that of the P${}_{\beta }$/AP${}_{\alpha }$. However, the P${}_{\alpha }$ /AP${}_{\beta }$ state is blocked while the P${}_{\beta }$/AP${}_{\alpha }$ state is conducting within the bias range from −0.1 to −0.3 V. The observed spin filtering effect, characterized by $SFE=[\left(I_{\alpha }-I_{\beta }\right)/\left(I_{\alpha }+I_{\beta }\right)]\times 100\%$ [47], ranges from nearly 100% to nearly −100% within the bias voltage range. Therefore, device M1 exhibits an exceptional near-perfect *dual spin filtering effect*. Compared to the STGNR (5, 3) device with pristine electrodes [21], the B edge-doped STGNR electrode of M1 greatly enhances the overall transport currents (>0.7 μA) and strengthens the dual spin filter efficiency in the P configuration.

Secondly, all devices exhibit significant spin-rectifying effect. The nonlinear and unidirectional spin-polarized current for the P${}_{\alpha }$/AP${}_{\beta }$ electron of device M1 can be observed in Figure 2a under positive bias but suppressed under the negative bias, while for the P${}_{\beta }$/AP${}_{\alpha }$, the opposite behaviors are observed. To evaluate the rectifying effect, the spin-resolved RR of device M1 is presented in Figure 2b, i.e., $RR_{\sigma }\left(V\right)=|I_{\sigma }\left(V\right)-I_{\sigma }\left(-V\right)|/|Min\left\{I_{\sigma }\left(V\right),I_{\sigma }\left(-V\right)\right\}|$ [35]. It can be seen that giant RR can be achieved in device M1 and the maximum value of RR reaches 4.5 ×$10^{5}$ and 1.9 ×$10^{6}$, 3.0 ×$10^{5}$ and 9.3 ×$10^{4}$ for the P${}_{\alpha }$ and AP${}_{\beta }$, P${}_{\beta }$ and AP${}_{\alpha }$ channels, respectively. The M1 device structure is thus a high-performance spin diode. For device M2 (Figure 2c), a current peak appears under the negative bias voltages of −0.6 ∼−0.2 V for all spin-resolved transport channels. The current is, however, suppressed under positive bias. The maximum RR of device M2 reaches above 10${}^{2}$ (see Figure 2d). This suggests that the device M2 exhibits inverse rectification and could be operated as a molecular spin diode and random access memory cells. As shown in Figure 2e, the device M3 shows semiconducting behavior with a threshold voltage window around −0.3 ∼ 0.1 V. The device M3 exhibits a rectification effect with a maximum ratio around 10${}^{3}$ for α electrons, as shown in Figure 2f.

Thirdly, strong NDR behaviors are evident for both devices M1 and M2. The peak-to-valley ratio of the NDR effect, defined as $PVR=I_{peak}/I_{valley}$ [23], is about 7.1 × 10${}^{5}$ for the P${}_{\beta }$ of Device M1 and 2.1 × 10${}^{3}$ for the P${}_{\alpha }$ of device M2. Such NDR with large PVR can be highly beneficial for applications in electrically induced fast switching and in computing electronic devices.

### 3.2. Electronic Band Structures of the Electrodes and Spin-Dependent Transmission Spectra

**Figure 3:**
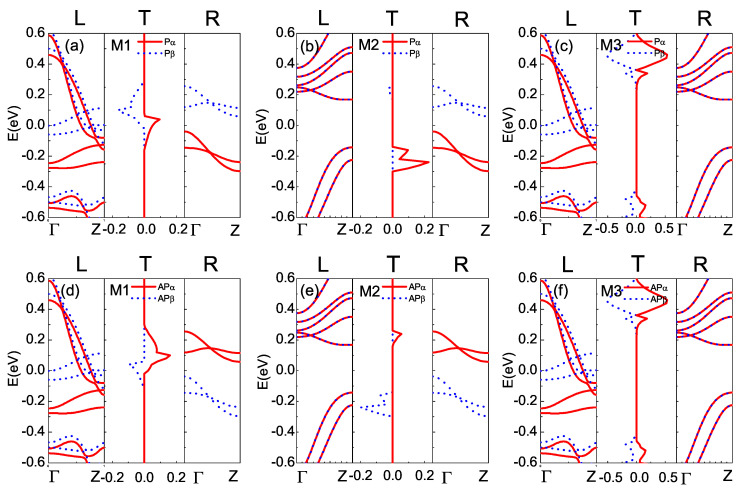
(Color online) The relation of the spin-dependent transmission spectrum (T), left electrode (L) and right electrode (R) band structures for M1-M3 at zero bias. (**a**–**c**) For P configuration. (**d**–**f**) For AP configuration. The red (blue) color denotes α-spin (β-spin) states and Fermi energy E${}_{F}$ is set to zero.

To explain the above I-V characteristics, the spin-dependent transmission (T) spectra and the spin-resolved band structure for the left (L) and right (R) electrode are calculated in Figure 3. Here, Figure 3a–c correspond to the P configuration, and Figure 3d–f correspond to the AP configuration for M1–M3, respectively. The red and blue lines denote the spin-up (α-spin) and spin-down (β-spin) states. The computed Fermi energy E${}_{F}$ are −5.85 eV, −1.73 eV and −5.85 eV for M1–M3, respectively, which are set to zero. Pristine STGNR (5, 3) is a bipolar magnetic semiconductor at the ground state [21,25]. In the P configuration for M1 and M2 Figure 3a,b, there are no subbands crossing the Fermi level (E${}_{F}$) for the pristine STGNR (5, 3) right electrode, and the the valance (α-spin) and conduction (β-spin) subbands are energetically split, which is in good agreement with the previous theoretical research [21]. Moreover, it can be seen from the left panels of Figure 2a that α- and β-spin subbands are separated and both cross the E${}_{F}$, thus revealing the metallic and spin-polarized electronic properties of the B-doped STGNR left electrode. The matching and the overlapping of valance (α-spin) and conduction (β-spin) bands in the B-doped left electrode and the pristine right electrodes contribute to two spin-polarized transmission peaks P${}_{\alpha }$ and P${}_{\beta }$ near the E${}_{F}$. However, from the left panels of Figure 3b, it can be seen that the N-doped STGNR left electrode in device M2 exhibits a semiconductor behavior with a band gap 0.31 eV. The energy bands of the N-doped left electrode and that of the pristine STGNR right electrodes are aligned with each other below and above the E${}_{F}$, which lead to a P${}_{\alpha }$ transmission peak situated below the E${}_{F}$ and a P${}_{\beta }$ transmission peak situated above the E${}_{F}$. In contrast, for the AP configurations Figure 2d,e, which can be achieved by an external perpendicular magnetic field that switches the spin ordering of the right electrode, the valance and conduction subband of the pristine STGNR right electrode is transformed into β-spin and α-spin, respectively. Therefore, the α-spin and β-spin transmission peaks in the AP configuration are interchanged as compared to that of the P configurations.

For device M3, the B-doped left electrode is metallic and the N-doped right electrode is semiconducting (see Figure 3c,f). The overlap of α- and β-spin subbands in both electrodes leads to two transmission peaks above and below the E${}_{F}$, respectively, but they do not contribute to electrical conduction at the low-bias regime due to the transmission gap around the E${}_{F}$. Figure 3 thus reveals that substitutional doping on the edges of STGNR electrodes can greatly influence the relative position and the height of the transmission peaks as the quantum transport of electrons across the device is mainly derived from the matching of the electronic band structure of the two electrodes so that band-to-band tunneling can occur to form a conduction current. In addition, the transmission spectra of M1-M3 are suppressed because the left and the right electrodes of STGNR are not identical, thus resulting in quantum destructive interference of the electron waves.

### 3.3. The Molecular Projected Self-Consistent Hamiltonian(MPSH), Spin Polarized Density, and Transmission Pathway

**Figure 4:**
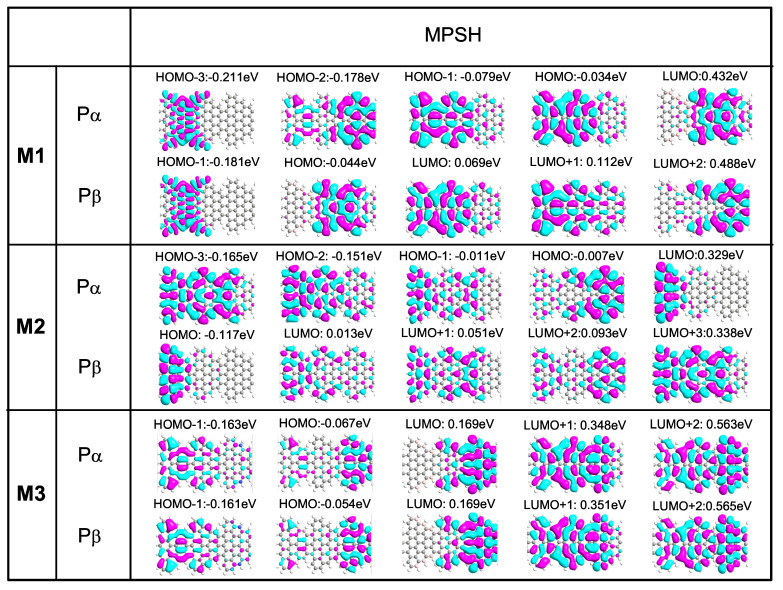
(Color online) The spatial distribution of the MPSH for M1-M3 at zero bias in P configuration.

The transmission behavior can also be understood from the energy level and the degree of delocalization of the frontier molecular orbitals (FMOs), especially the highest occupied molecular orbital (HOMO) and lowest unoccupied molecular orbital (LUMO) which are near to the Fermi level. A more delocalized molecular projected self-consistent Hamiltonian (MPSH) distribution of FMOs may contribute to greater transmission probability [48]. To further understand the origin of different transport behaviors of devices M1-M3, we show the energy level and spatial distribution of FMOs around E${}_{F}$ for with P configuration at zero bias in Figure 4. In general, there are three delocalized HOMOs (HOMO, HOMO-1 and HOMO-2) and two LUMOs (LUMO, LUMO+1) for P${}_{\alpha }$/P${}_{\beta }$ channels of device M1, which contribute to the P${}_{\alpha }$ and P${}_{\beta }$ transport peaks, respectively. Thus, the M1 structure is a magnetic conductor. However, for M2, although the N-doped left electrode is a nonmagnetic semiconductor, the M2 structure inherits the magnetism of the right STGNR electrodes, thus resulting in a magnetic semiconductor characteristic. Figure 4 also suggests that the delocalized two HOMOs (HOMO-2 and HOMO-3) for α spin channels lead to the corresponding P${}_{\alpha }$ transmission peaks located below the E${}_{F}$. For β spin channels, there is only a slight delocalized LUMO+3 contributed to the small transmission peak above the E${}_{F}$. In addition, the delocalized nature of the LUMOs(LUMO+1 and LUMO+2) of P${}_{\alpha }$ and P${}_{\beta }$ channel contribute to the significant transmission peaks of device M3 above the E${}_{F}$. This leads to the semiconductor behavior of M3.

**Figure 5:**
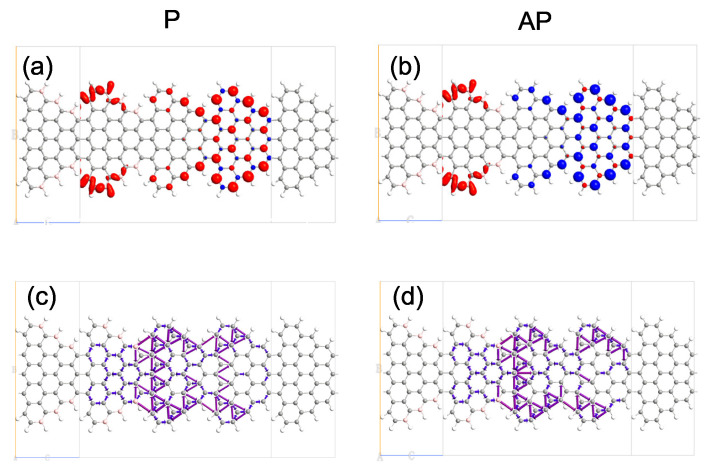
(Color online) (**a**,**b**) Isosurface plots of the spin density ($\nabla \rho =\rho_{\alpha }-\rho_{\beta }$) for M1 in P/AP configuration, where values for red (α-spin) and blue (β-spin) isosurfaces are ±0.002∣e∣/Å^3^, respectively. (**c**,**d**) The transmission pathway of the central scattering region for M1 in P/AP configuration, respectively.

Previous work has predicted the existence of a spin parallel coupling between both edges of the ferromagnetic STGNR and the spin-polarized electrons that are conducting at both edges [21,25]. Therefore, substitutional edge-doping can significantly influence the band structures of STGNR electrodes and thus can be employed to engineer the electronic transport properties of the nanostructure. To investigate the edge-doping effects, we study the spin electron density ($\nabla \rho =\rho_{\alpha }-\rho_{\beta }$) and the transmission pathway of the central scattering region using M1 as an illustrative example. The isosurface plots of the spin electron density for M1 in P and AP configurations are shown in Figure 5a,b, where $\rho_{\alpha }$ and $\rho_{\beta }$ denote the electron density of α-spin (red) and β-spin (blue), respectively. As shown in Figure 5a,b, all the carbon atoms display spin-polarized states in the pristine STGNR (5, 3) right electrodes with parallel spin coupling between both edges, which is consistent with previous investigations [21,25]. While for the B-doped STGNR left electrode, the spin polarization states are mainly localized at the doped edge carbon atoms, which cause electronic and magnetic structures of B-doped STGNR (5, 3) to be rather different from the pristine STGNR (5, 3) and influence the electronic transport properties of M1. In addition, the transmission pathway of the scattering central region for M1 in P/AP configuration are shown in Figure 5c,d, respectively. The arrows indicate the possible transmission pathway of the electrons. Our calculations suggest that electrons mainly transport among carbon atoms at both edges in the pristine STGNR right electrodes. However, for the B-doped STGNR left electrode, electrons mainly transport among carbon atoms in the middle of the ribbons because of the influence of B-doping region.

### 3.4. Electron Energy and Bias Voltage Dependence of the Spin-Resolved Transmission Spectrum

**Figure 6:**
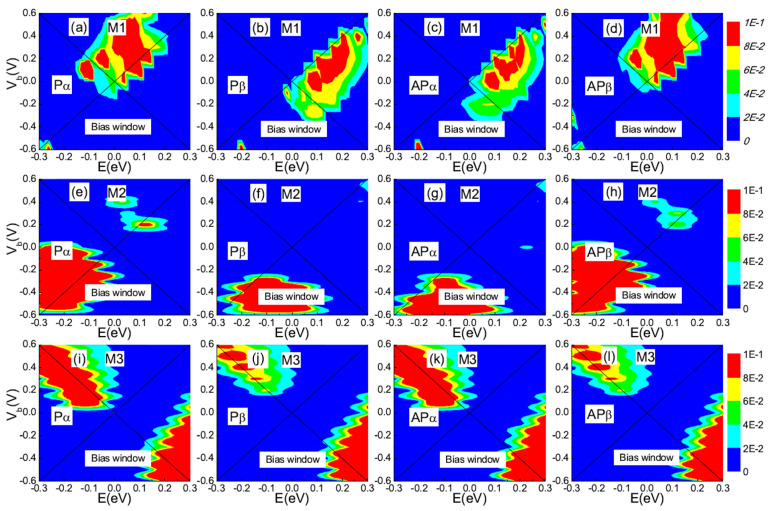
(Color online) 3D plot of the spin-resolved transmission spectra on the E-V${}_{b}$ plane, where (**a**–**d**) corresponds to M1 in P/AP configuration, respectively. (**e**–**h**) and (**i**–**l**) The same as (**a**–**d**) for M2 and M3, respectively. The (black) cross line region is referred to as the bias window.

According to the Landauer–Büttiker formula, the spin-dependent current is determined by the transmission within the bias window (BW). To further explain the I-V characteristics, we present the 3D plot of the spin-resolved transmission spectra as a function of both electron energy (E) and bias (V${}_{b}$) for devices M1-M3 in Figure 6. The region highlighted with the (black) cross line represents the BW. By comparing Figure 6a,c with Figure 6b,d, it is can be seen that there is significant P${}_{\alpha }$ and AP${}_{\beta }$ transmission for device M1 under the bias regime V${}_{b}>$ 0 V, while the step edge of the P${}_{\beta }$ and AP${}_{\alpha }$ transmission shifts to the higher-energy regime and remains nearly outside of the bias window. Thus, the P${}_{\alpha }$ and AP${}_{\beta }$ transport channels are in the ON state when V${}_{b}>$ 0 V while the P${}_{\beta }$ and AP${}_{\alpha }$ transport channels are in the OFF state. On the contrary, the P${}_{\beta }$ and AP${}_{\alpha }$ transmission spectra are driven to a lower energy and move into the transport window within the bias range of (−0.4, 0 V), while the P${}_{\alpha }$ and AP${}_{\beta }$ transmissions are almost zero within this bias. As a result, the P${}_{\alpha }$ and AP${}_{\beta }$ transport channel is OFF while the P${}_{\_}\beta$ and AP${}_{\_}\alpha$ transport channel is ON. This leads to a significant difference in the tunneling current between the two spin states, as shown in Figure 2a, and directly leads to the near-perfect dual spin filtering effect as well as the strong spin rectification effect. Moreover, the asymmetry of the I-V characteristic of devices M2 and M3 can also be understood by analyzing the spin-resolved transmission spectra as a function of bias. As shown in Figure 6e–h, the spin-up and spin-down transmission spectrum fill the negative bias (−0.6, −0.2 V), which contributes to the spin-resolved currents under negative bias voltages and thus the inverse rectification effect. For M3, as shown in Figure 6i–l, the spin-dependent transmission peaks occur within the negative bias (−0.6, −0.3 V) and the positive bias (0.2, 0.6 V), while they are far away from BW within the bias (−0.2, −0.1 V). Thus, it leads to a significant asymmetric current as shown in Figure 2e.

**Figure 7:**
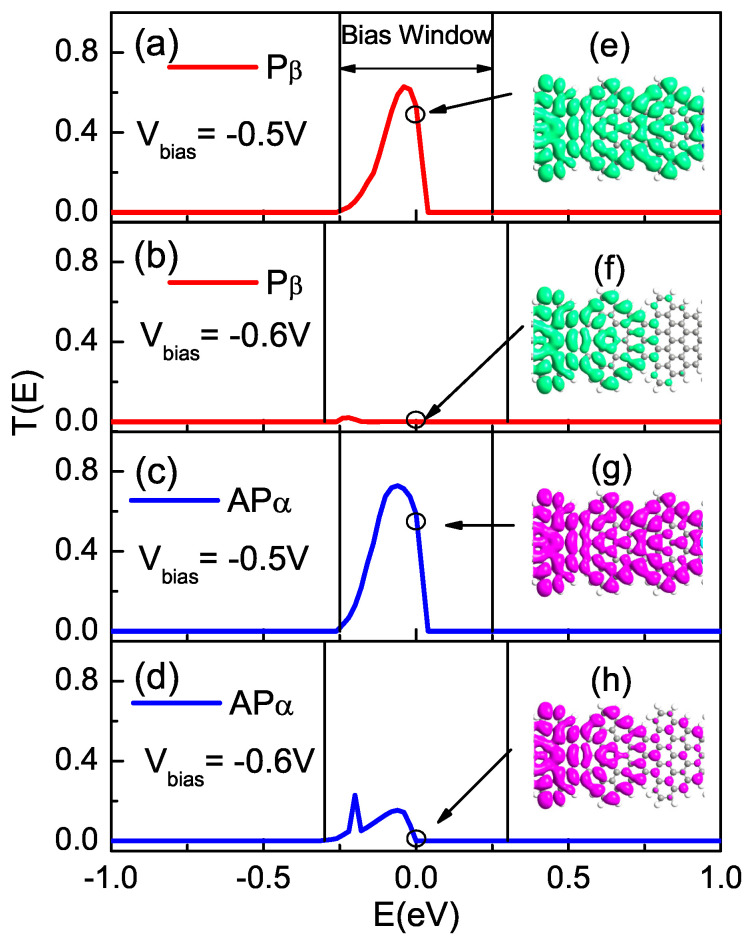
(Color online) Transmission spectra for P${}_{\beta }$/AP${}_{\alpha }$ state under the applied bias (**a**,**c**) −0.5 and (**b**,**d**) −0.6 V for model M2, respectively. Inset: spin-resolved LDOS at $E_{F}$, (**e**,**f**) for P${}_{\beta }$ state and (**g**,**h**) for AP${}_{\alpha }$ state. The region between the black lines is the bias window.

Finally, it is known that the NDR effect may arise when the conduction orbital is suppressed at a certain bias voltage [49]. Here, we use device M2 as an illustrative example to explain the origin of the observed NDR behavior. In Figure 7, we present the spin-resolved transmission associated with local density of states (LDOS) around E${}_{F}$ at two special bias voltages of −0.5 and −0.6 V for device M2. Clearly, as shown in Figure 7a [inset (e)] and Figure 7c [inset (g)], when the bias voltage is −0.5 V, there is a large P${}_{\beta }$ and AP${}_{\alpha }$ transmission peak within the BW, which leads to a large P${}_{\beta }$ and AP${}_{\alpha }$ current, respectively. Furthermore, the transport mechanism originates from the difference in the LDOS around E${}_{F}$. The π-orbital amplitude is spatially delocalized throughout the scattering region under bias −0.5 V, which leads to big transmission peaks of P${}_{\beta }$ and AP${}_{\alpha }$ electrons within the bias window (denoted by green and purple in Figure 7, respectively). However, when the bias increases to −0.6 V, the electronic cloud is principally localized on the left electrode region, as shown in Figure 7b [inset (f)] and Figure 7d [inset (h)], which results in the suppression of the P${}_{\beta }$ and AP${}_{\alpha }$ orbital and the corresponding transmission peaks shift away from the E${}_{F}$ and become smaller under the bias −0.6 V. As a result, the current decreases under higher bias and the NDR effect emerges.

We briefly discuss the spin flip process, which is expected to influence the electron and spin transport of GNR nanodevice. As demonstrated in a previous ab initio transport simulation [50], the spin-flip process is very weak in a clean GNR nanodevice, but such a process can be significantly enhanced in the presence of hydrogen adsorbates in the transport channel. We thus expect an impurity-induced spin-flip process to generate rich transport signatures and may even be employed to engineer the transport properties of a GNR-based device. The spin-flip effects in the STGNR nanodevice shall be an interesting topic to explore in future works. Finally, we would like to point out that when phonon scattering processes are taken into account, such as the inelastic case, the valley current may be significantly increased, thus resulting in the peak-to-valley ratio being decreased by several orders of magnitude when compared to the ballistic transport assumption [51]. Therefore, phonon scattering is expected to play a significant role, especially in nanodevices with a longer channel length in which the ballistic transport assumption is no longer valid.

## 4. Conclusions

In summary, we have proposed a graphene-based spintronics molecular nanodevice by adopting the pristine and B/N edge-doped STGNR (5, 3) as electrodes. The edge doping of B, N can significantly affect the transport properties of these systems. Interestingly, the dual spin filtering effect with an SFE of nearly 100%, high-performance spin rectifier with the RR up to about 1.9 × $10^{6}$ and strong NDR behavior with the PVR up to about 7.1 × 10${}^{5}$ can be achieved by a B/N edge-doped electrode. By analyzing the spin-resolved transmission spectra and the band matching relation between two electrodes, it is found that the substitutional edge-doping significantly influences the band structure of STGNR electrodes and can be employed to engineer the relative position and height of the transmission peaks by matching the band structure of the two electrodes. The perfect dual spin filter and spin diode effect are due to the unique band overlap pattern for the pristine and B/N edge-doped STGNR (5, 3) electrode, as well as the evolution of transmission coefficients with the bias voltages and electron energies. Finally, the NDR behaviors are found to originate from the suppression of the conduction orbital at certain bias voltages. These findings reveal the potential of the proposed graphene-based nanodevices as building blocks of novel spintronic device technology, such as a highly effective spin-filter, spin-diode and spin-switching device.

## Data Availability

The data is available on reasonable request from the corresponding author.
